# Evaluation of nanoparticle albumin-bound paclitaxel loaded macrophages for glioblastoma treatment based on a microfluidic chip

**DOI:** 10.3389/fbioe.2024.1361682

**Published:** 2024-03-18

**Authors:** Zuorun Xie, Junyi Ye, Xinghua Gao, Hang Chen, Maosong Chen, Jiangfang Lian, Jingyun Ma, Hongcai Wang

**Affiliations:** ^1^ Department of Neurosurgery, The Affiliated Lihuili Hospital of Ningbo University, Ningbo, Zhejiang, China; ^2^ Materials Genome Institute, Shanghai University, Shanghai, China; ^3^ Ningbo Institute of Innovation for Combined Medicine and Engineering, The Affiliated Lihuili Hospital of Ningbo University, Ningbo, Zhejiang, China

**Keywords:** microfluidic chip, glioblastoma, macrophage-based carrier, drug delivery, nanoparticle albumin-bound paclitaxel

## Abstract

**Introduction:** Glioblastoma (GBM) is a primary brain malignancy with a dismal prognosis and remains incurable at present. In this study, macrophages (MΦ) were developed to carry nanoparticle albumin-bound paclitaxel (nab-PTX) to form nab-PTX/MΦ. The aim of this study is to use a GBM-on-a-chip to evaluate the anti-GBM effects of nab-PTX/MΦ.

**Methods:** In this study, we constructed nab-PTX/MΦ by incubating live MΦ with nab-PTX. We developed a microfluidic chip to co-culture GBM cells and human umbilical vein endothelial cells, mimicking the simplified blood-brain barrier and GBM. Using a syringe pump, we perform sustainable perfusion of nutrient media. To evaluate the anti-GBM effects nab-PTX/MΦ, we treated the GBM-on-a-chip model with nab-PTX/MΦ and investigated GBM cell proliferation, migration, and spheroid formation.

**Results:** At the chosen concentration, nab-PTX did not significantly affect the viability, chemotaxis and migration of MΦ. The uptake of nab-PTX by MΦ occurred within 1 h of incubation and almost reached saturation at 6 h. Additionally, nab-PTX/MΦ exhibited the M1 phenotype, which inhibits tumor progression. Following phagocytosis, MΦ were able to release nab-PTX, and the release of nab-PTX by MΦ had nearly reached its limit at 48 h. Compared with control group and blank MΦ group, individual nab-PTX group and nab-PTX/MΦ group could inhibit tumor proliferation, invasion and spheroid formation. Meanwhile, the anti-GBM effect of nab-PTX/MΦ was more significant than nab-PTX.

**Discussion:** Our findings demonstrate that nab-PTX/MΦ has a significant anti-GBM effect compared to individual nab-PTX or MΦ administration, suggesting MΦ as potential drug delivery vectors for GBM therapy. Furthermore, the developed GBM-on-a-chip model provides a potential *ex vivo* platform for innovative cell-based therapies and tailored therapeutic strategies for GBM.

## 1 Introduction

Glioblastoma (GBM) is recognized as the foremost primary brain tumor in terms of aggressiveness and prevalence, presenting challenges in achieving completely surgical resection and usually exhibiting a propensity for recurrence (Ostrom et al., 2022; Qi et al., 2023). The blood-brain barrier (BBB) poses a formidable obstacle, impeding the penetration of chemotherapeutic agents into brain tissue and thereby restricting their efficacy against GBM (Wu et al., 2023). Consequently, overcoming the BBB and effectively delivering drugs to GBM tumors are imperative for the successful treatment of this condition.

Macrophages (MΦ), a subtype of phagocytes derived from leukocytes, inherently possess the ability to cross the BBB and migrate naturally to tumor tissues. They exhibit a robust capacity for uptake and internalization, with their intracellular space enabling drug storage within the cytoplasm (Sharma et al., 2021). Consequently, this particular characteristic offers notable benefits in the context of cancer-targeted therapies. GBM can secrete colony stimulating factors, which in turn attract the infiltration of tumor associated MΦ and thereby facilitate the continuous recruitment of circulating MΦ (Nance et al., 2022). Thus, one strategy to improve drug delivery across the BBB is to load nanoparticles into live MΦ through incubation, taking advantage of MΦ’s natural uptake capacity and the passive diffusion properties of drugs (Hou et al., 2020; Zhang et al., 2022). Furthermore, MΦ play a substantial role in the regulation of the immune microenvironment of tumors. M1-phenotype MΦ exhibit tumoricidal properties and promote inflammation, whereas M2-phenotypr MΦ possess pro-tumoral characteristics and exert anti-inflammatory effects (Kerneur et al., 2022; Chen et al., 2023). Previous studies have indicated that converting M2-phenotype MΦ into M1-phenotype MΦ and inhibiting MΦ M2 polarization are frequently employed strategies to suppress tumor growth (Dong et al., 2020; Han et al., 2023).

Paclitaxel (PTX) is a chemotherapeutic agent with a significantly higher potency (approximately 1,400 times) compared to temozolomide (the first-line chemotherapy agent for GBM) (Zhang et al., 2020). However, PTX is unable to penetrate the BBB in contrast to temozolomide (Kalynovska et al., 2020). After intravenous administration, nab-PTX exhibits poor penetration into the central nervous system, posing challenges in effectively treating GBM in clinical trials (Sonabend et al., 2023). The commercially available nanoparticle albumin-bound paclitaxel (nab-PTX) is created by binding paclitaxel to albumin, forming a complex that enhances solubility and stability. This formulation, also known as Paclitaxel for Injection (Albumin Bound), has demonstrated the ability to enhance the anti-tumor effectiveness of PTX while simultaneously reducing the associated side effects (Fan and He, 2022). The expression of albumin-binding proteins such as SPARC and gp60, was observed to be upregulated on both GBM cells and the endothelial cells of tumor neovasculature (Lin et al., 2016; Li et al., 2023). In GBM, gp60 may interact with SPARC to regulate the uptake and mechanisms of action of albumin-bound paclitaxel (nab-PTX), thereby affecting its therapeutic efficacy. Consequently, the albumin component of nab-PTX can enhance the tumor penetration. Moreover, it can facilitate cellular uptake through transport mechanisms mediated by SPARC and gp60. Additionally, PTX has been found to mimic LPS in activating the NF-κB pathway and exerting immunomodulatory effects by promoting MΦ M1 polarization (Yamaguchi et al., 2017; Chen et al., 2021).

In recent years, three-dimensional (3D) cell culture models have been extensively used as *in vitro* models for drug evaluation and cancer research. One promising methodology involves the use of microfluidic technology to create 3D tumor spheroids, which are small and compact cell aggregates consisting of cancer cells (Ko et al., 2019). Compared to traditional two-dimensional monolayer cell culture, 3D cell culture allows for cell growth and interactions with the surroundings in all directions, leading to the formation of tumor spheroids that more accurately mimic the structure and behavior of tumors *in vivo* (Samiei et al., 2020). Additionally, the utilization of microfluidic devices offers the advantage of precise control over the cellular environment, including the regulation of nutrient and oxygen flow. This capability enables the creation of a more realistic representation of a solid tumor characterized by a gradient of nutrients, metabolites, and oxygen across its radius (Yi et al., 2019).

**FIGURE 1:**
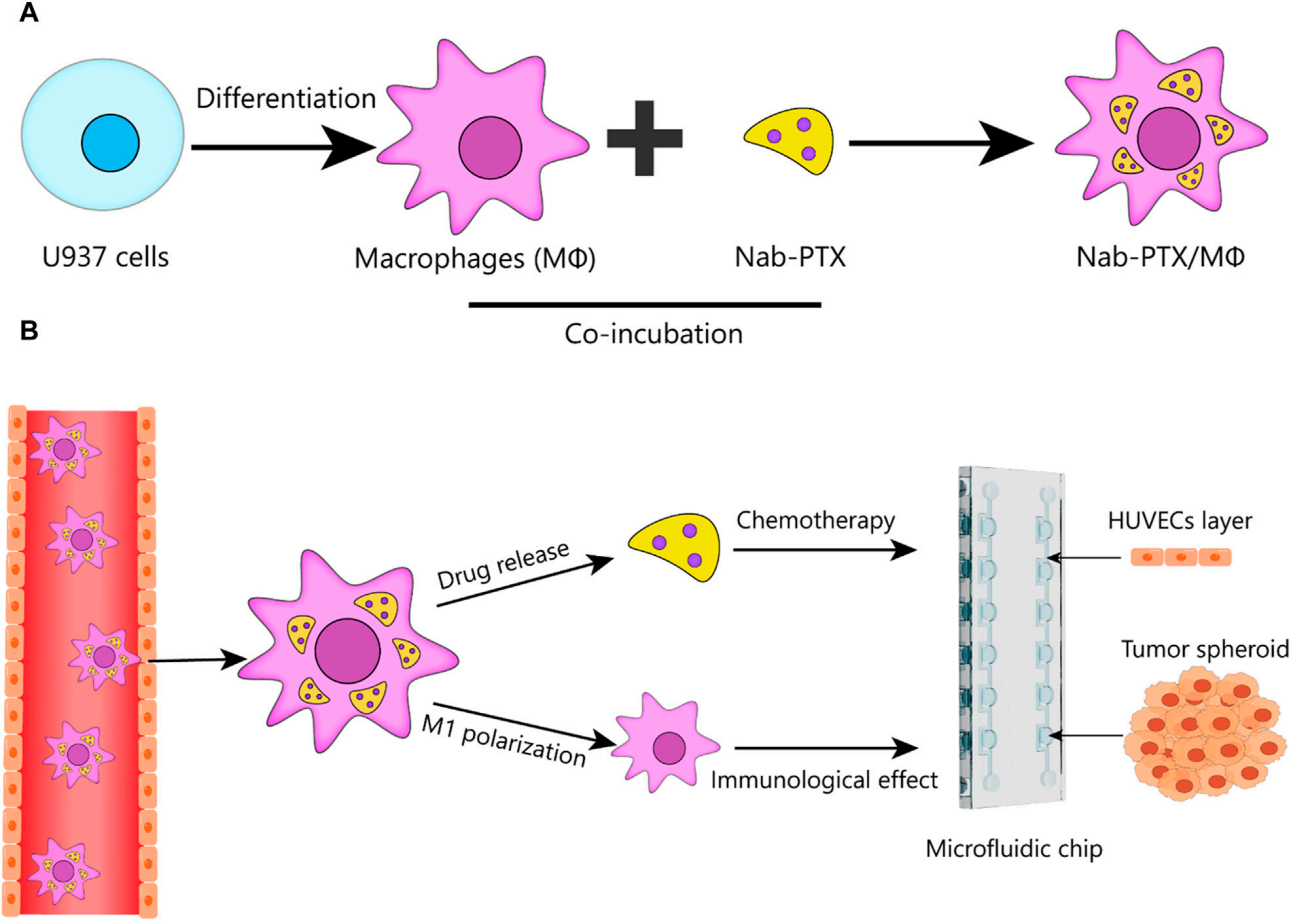
Schematic design of nab-PTX loaded MΦ for administration through the BBB and tumor targeting to enhance the therapeutic efficiency of GBM. **(A)** Preparation of nab-PTX/MΦ. **(B)** Schematic that shows the ability of nab-PTX/MΦ to transmigrate across the BBB and reach the tumor spheroid, where they can exert both chemotherapeutic and immunotherapeutic effects to produce an anti-tumor response.

**FIGURE 2:**
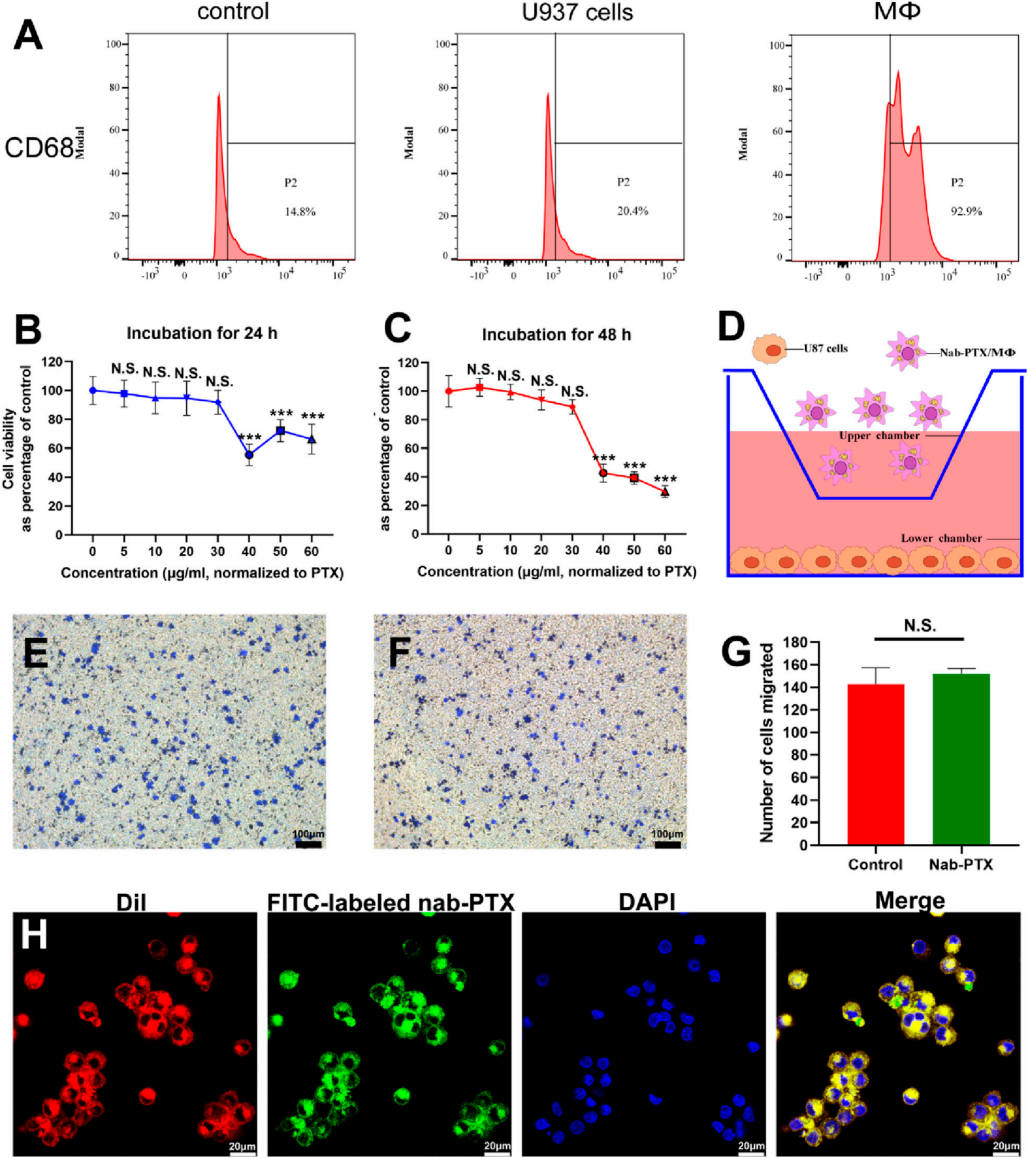
U937 cells differentiated into MΦ and the characterization of nab-PTX/MΦ. **(A)** The expression levels of CD68 in U937 cells and MΦ were determined by flow cytometry analysis. **(B,C)** Cytotoxicity of nab-PTX towards MΦ for 24 h and 48 h. ** and *** represent *p* < 0.01 and *p* < 0.001 versus the 0 μg/mL group, respectively. **(D–G)** Transwell migration assay. **(D)** Schematic illustration of the transwell model for the chemotaxis and migration of nab-PTX/MΦ towards GBM cells. **(E)** Transwell migration assay of blank MΦ **(F)** Transwell migration assay of nab-PTX/MΦ. Scale bars represent 100 μm. **(G)** Quantification of migrated cells. **(H)** The encapsulated nab-PTX was uniformly dispersed within MΦ and observed by LSCM. Red fluorescence comes from cell membranes, green fluorescence comes from FITC-labeled nab-PTX, and blue fluorescence represents nuclear staining. Scale bars represent 20 μm.

Here, we developed a bioengineered model of GBM in a microfluidic chip and perform the first systematic analysis of the anti-GBM effect of nab-PTX/MΦ using this 3D microfluidic model. This standardized microfluidic platform, constructed from polydimethylsiloxane (PDMS), offers easy accessibility for all researchers without the need for complicated pre-treatment procedures. Using this platform, we generate GBM spheroids within chambers and co-culture them with human umbilical vein endothelial cells (HUVECs) on the periphery of the chamber to mimic the BBB. This study aims to achieve two main objectives: 1) to construct a live MΦ-based nab-PTX delivery system for nab-PTX and to validate that the nab-PTX/MΦ exhibited M1 phenotype (Figure 1A); 2) to investigate the anti-GBM effect of nab-PTX/MΦ by assessing their impact on reducing cell proliferation, migration, and spheroid formation in GBM cells using the developed microfluidic chip (Figure 1B).

## 2 Materials and methods

### 2.1 Cell culture

Both U87-MG and U87-eGFP cells were gifted from Dr. Xu, which were purchased from iCell Bioscience Inc. (Shanghai, China) and were cultured in DMEM medium. U937 cells were purchased from iCell Bioscience Inc. (Shanghai, China) and cultured in RPMI 1640 medium (VivaCell, Shanghai, China). Subsequently, U937 cells were treated with a concentration of 0.1 μg/mL Phorbol-12-myristil-13-acetate (PMA; GlpBio, Shanghai, China) for 24 h, resulting in their differentiation into MΦ. The intracellular flow cytometry protocol was employed to detect the presence of CD68. Following fixation with a fix buffer (Proteintech, Wuhan, China) and subsequent washing with a perm buffer (Proteintech), the cells were incubated with a concentration of 1 μg/mL anti-human CD68-fluorescein isothiocyanate (FITC) obtained from Proteintech. The stained cells were then subjected to analysis using a flow cytometer (Beckman Coulter, Suzhou, China). HUVECs were obtained from iCell Bioscience Inc. and cultured in DMEM medium (VivaCell). All the media were supplemented with 10% fetal bovine serum (VivaCell) and 1% penicillin-streptomycin (Gibco, Shanghai, China). All cells were cultured in a humidified incubator at a temperature of 37°C with a 5% CO_2_ environment.

### 2.2 Cytotoxicity of nab-PTX towards MΦ and preparation of Nab-PTX/MΦ

Nab-PTX was purchased from Jiangsu Hengrui Medicine Company (Jiangsu, China). The blank MΦ (untreated MΦ) were uniformly seeded into 96-well plates (1 × 10^4^ cells per well). Different concentrations of nab-PTX (0, 5, 10, 20, 30, 40, 50, 60 μg/mL) were respectively added into the wells. MΦ were incubated with nab-PTX for 24 and 48 h. Subsequently, the cell viability of MΦ was assessed using the Cell Counting Kit-8 (CCK-8; GlpBio).

Nab-PTX/MΦ were formed by incubating MΦ with nab-PTX. Nab-PTX and 5 ×10^6^ MΦ were seeded into the sterile tube. MΦ were cultured with a concentration of 30 μg/mL nab-PXT for 6 h, followed by three washes with phosphate buffer solution (PBS; VivaCell). The resulting nab/PTX suspension was then immediately utilized for the subsequent experiment.

### 2.3 The uptake of PTX in Nab-PTX/MΦ and release of PTX from nab-PTX/MΦ

MΦ were cultured in individual laser confocal culture dishes and incubated with FITC-labeled nab-PTX at a concentration of 30 μg/mL for various durations (0, 1, 2, 4, 6, 8, 12 h). After incubation, the cells were washed with PBS to remove any unbound nab-PTX. The cells were fixed with 4% paraformaldehyde (PFA; VivaCell) for 15 min, followed by staining with DAPI (Beyotime, Shanghai, China) to facilitate visualization of the nuclei. The resulting samples were subsequently observed using a Leica SP8 laser scanning confocal microscopy (LSCM; Leica, Wetzlar, Germany). The images were analyzed using Image J software. By comparing the fluorescence density of MΦ treated with FITC-labeled nab-PTX for various durations, the extent of nab-PTX uptake by MΦ was assessed. In order to quantify the uptake amount of PTX in Nab-PTX/MΦ, Nab-PTX/MΦ were lysed by a RIPA cell lysis buffer (NCM Biotech, Suzhou, China), and PTX was subsequently released from MΦ. The resulting cell lysate was then centrifuged at 8,000 rpm for 5 min, and the supernatant was collected. Methanol was added to the supernatant, which was then vortex mixed and centrifuged at 8,000 rpm for 10 min. The amount of PTX in the supernatant was measured using ultra performance liquid chromatography tandem mass spectroscopy (UPLC-MS). The analyses were performed on a Waters ACQUITY H-Class Liquid Chromatograph/SQD2 Mass Spectrometer (Milford, MA, United States).

FITC-labeled nab-PTX/MΦ were generated by incubating MΦ with 30 μg/mL FITC-labeled nab-PTX in the laser confocal culture dish for a duration of 6 h. Following incubation, the cells were washed with PBS to remove any unbound nab-PTX. Subsequently, the cells in separate culture dish were incubated with 1,640 medium for various durations (12, 24, 48, 72 h). Nab-PTX/MΦ were visualized using LSCM. To quantify the released amount of PTX from Nab-PTX/MΦ, nab-PTX/MΦ (1×10^6^) were seeded in the culture dish. At predetermined time intervals, the supernatant medium from each culture dish was collected, and the total amount of released PTX in the medium was determined using UPLC-MS.

### 2.4 Chemotactic function and migration ability of nab-PTX/MΦ

MΦ (1 × 10^6^ cells/mL) were initially loaded with 30 μg/mL nab-PTX to generate nab-PTX/MΦ. GBM cells were inoculated into the lower chamber of 24-well transwell plates at a density of 1 × 10^5^ cells per well. Blank MΦ or nab-PTX/MΦ were seeded into the upper chamber at a density of 5 × 10^4^ cells per well. Following a 24 h of incubation period, the transwell chambers were fixed with 4% PFA for 15 min and subsequently stained with 0.1% crystalline violet (Solarbio, Beijing, China) for 15 min. Five random visual fields per chamber were selected to count the cells that migrated the lower chamber.

### 2.5 Mixed culture of nab-PTX/MΦ and GBM cells

Initially, the MΦ were incubated with FITC-labeled nab-PTX for 6 h. Subsequently, FITC-labeled nab-PTX/MΦ (1 × 10^4^ cells) were mixed-cultured with GBM cells (1×10^4^ cells) in a confocal dish under regular culture conditions for 24 h. The mixed culture was visualized using a LSCM after DAPI nuclear counterstaining and fixation with 4% PFA.

### 2.6 *In Vitro* cytotoxicity of nab-PTX/MΦ

After incubation for 48 h, the supernatant of nab-PTX/MΦ (1 × 10^6^) was collected. The equal amount of nab-PTX (equivalent to 6 μg/mL PTX) and the supernatant of blank MΦ incubated for 48 h were set as control groups. U87 cells were seeded on a 96-well plate at a density of 1 × 10^4^ cells per well, and different formulations were applied to the tumor cells and incubated for 48 h. Cell viability was assessed by CCK-8.

### 2.7 Assay of the change in MΦ phenotype

Lipopolysaccharides (LPS; GlpBio) was used to induce MΦ M1 polarization (Zhang et al., 2023). Subsequently, the M1 polarization of MΦ in three groups (blank MΦ, MΦ incubated with nab-PTX, MΦ incubated with LPS) was compared (Supplementary Figure S2).

First, the Flow cytometry assay was employed detect CD86 using an extracellular flow cytometry protocol. The cells were fixed with a fix buffer and then incubated with anti-human CD86-PE (Proteintech) for 30 min at 4°C in the absence of light. The stained cells were analyzed using the flow cytometer.

Second, the Immunofluorescence assay was also used to detect CD86. MΦ were fixed in 4% PFA for 15 min, followed by incubation in a 5% bovine serum albumin solution for 1 h. Subsequently, the cells were incubated with the primary antibody: rabbit anti-human CD86 (1:100 dilution; Zenbio, Chengdu, China) overnight at 4°C. The secondary antibody, goat anti-rabbit IgG (1:100 dilution; Zenbio), was then applied for 1 h. DAPI was used at a concentration of 100 ng/mL to visualize the nuclei.

Third, Quantitative real-time PCR assay was performed for the relative gene expression analysis. Total RNA was extracted from three groups (blank MΦ, MΦ incubated with nab-PTX, MΦ incubated with LPS) using the TransZol Up RNA Kit (TransGen Biotech, Beijing, China) and converted to cDNA using the EasyScript^®^ All-in-One First-Strand cDNA Synthesis SuperMix for qPCR Kit (TransGen Biotech, Beijing, China). Then, quantitative RT-PCR was carried out by PerfectStart^®^ Green qPCR SuperMix Kit (TransGen Biotech, Beijing, China). Primer sequences are demonstrated in Table 1. Assays were made in triplicate, and the relative gene expression levels were quantified by using the ∆∆CT (cycle threshold) method.

**TABLE 1:** List of primer sequences of human used in RT-qPCR.

Gene	Forward primer (5′-3′)	Reverse primer (5′-3′)
GADPH	CAGCCCCAGCGTCAAAGG	GCT​CTC​CAG​AAC​ATC​ATC​C
TNF-α	AAC​TAC​AGA​CCC​CCC​CTG​AAA​AC	AAG​AGG​CTG​AGG​AAC​AAG​CAC​C
IL-1β	ACG​CTC​CGG​GAC​TCA​CAG​CA	TGA​GGC​CCA​AGG​CCA​CAG​GT
IL-6	TTCACCAGGCAAGTCTCC	ATA​CTC​GAC​GGC​ATC​TCA​G

### 2.8 Design and construction of the microfluidic device

Microfluidic devices were fabricated using standard soft- and photo-lithography techniques. Master templates were produced by applying SU-8 photoresist (MicroChem, United States) onto a silicon wafer, resulting in a final resist thickness of 150 μm. Polydimethylsiloxane (PDMS; Sylgard 184, Dow Corning, United States) was poured onto the silicon master at a ratio of 10:1 base to curing agent. The resulting mixture was then subjected to degassing in a vacuum desiccator for a duration of 20 min to eliminate any trapped air bubbles and cured at 80°C for a duration of 1 h. The PDMS devices were carefully peeled from the master mold and punched to create inlet and outlet ports. Finally, the PDMS devices were cleaned and irreversibly attached to glass microscope slides using oxygen plasma.

### 2.9 Cell loading and spheroid formation

Prior to cell culture, the microfluidic chip was subjected to sterilization through by injecting 75% ethanol and exposing it to high-intensity UV light. A diluent of Matrigel (Corning, Shanghai, China) in a ratio of 1:5 with DMEM, containing 5 ×10^6^ cells/mL, was introduced into the cell inlet using a micropipette. Subsequently, reverse injection of air was performed to remove the Matrigel diluent, resulting in the entrapment of the majority of the cells within the microwells. Following this, HUVECs were injected into the chip, and the chip was positioned upright and left undisturbed overnight. The cells were then incubated at a temperature of 37°C with 5% CO_2_ to facilitate the formation of spheroids.

HUVECs were analyzed by immunofluorescence staining for zonula occludens protein-1 (ZO-1) antibody on the designated culture day on chip to investigate the intercellular connection. Cells were fixed 4% PFA for 20 min, permeabilized using 0.5% Triton X-100 in PBS for 15 min, blocked with 5% BSA for 1 h, and then incubated with anti-rabbit primary ZO-1 antibody (AF5145, Affinity, Changzhou, China; 1:100 dilution in 2% BSA) overnight at 4°C. The following day, cells were incubated with FITC-tagged anti-rabbit secondary antibody (S0008, Affinity; 1:100 dilution in 2% BSA) for 1 h at room temperature. DAPI was used at a concentration of 100 ng/mL to visualize the nuclei. Finally, cells are visualized using a 63× oil immersion lens and single optical slices on LSCM.

### 2.10 Determination of anti-tumor effects of nab-PTX/MΦ in a GBM model

In order to ensure the availability of essential nutrients for cellular growth, the microfluidic chip was continuously perfused with nutrient media using a syringe pump (LSP02-2A, Longerpump, China). The culture medium in the syringes was separately supplemented with PBS, blank MΦ, nab-PTX, and nab-PTX/MΦ, divided into four groups for perfusion to the microfluidic chip. Following a 48-h incubation period, the viability of 3D multicellular tumor spheroids was assayed using Calcein-AM/PI Cell Live/Dead Assay Kit (Beyotime). Viable cells were identified using Calcein-AM (green), while non-viable cells selectively took up PI (red).

U87-EGFP cells, expressing green fluorescent protein (EGFP), were also employed to generate GBM spheroids for prompt and distinct assessment of the anti-GBM effects. The fluorescence emitted by these cells enables visualization and tracking of their behavior within the tumor microenvironment. By monitoring the fluorescence intensity or distribution of U87-EGFP cells over time, we can evaluate various aspects of tumor growth, invasion, and response to treatment. Following 48 h of diverse interventions, the U87-EGFP spheroids were observed using LSCM.

### 2.11 Statistical analysis

The data was analyzed using GraphPad Prism software and presented as the mean ± standard deviation (SD). The statistical significance of difference between groups was evaluated through One-way analysis of variance (ANOVA). Statistical significance was set at *p < 0.05, **p < 0.01 and ***p < 0.001.

## 3 Results

### 3.1 Differentiation of U937 monocytes into MΦ

U937 monocytes are a frequently utilized cell line for investigating macrophage differentiation (Nascimento et al., 2022). The most commonly used method to induce differentiation of U937 monocytes into MΦ is by treating them with PMA. This treatment effectively activates PKC and triggers various signaling pathways that facilitate macrophage differentiation (Khan et al., 2022; Shen et al., 2022; Tu et al., 2022). As depicted in Supplementary Figure S1, SU937 cells exhibited a spherical shape and lack adhesion, whereas PMA-induced MΦ exhibited a larger, more flattened morphology and formed compact colony-like structures that adhere strongly to the culture substrate. The expression of CD68, a specific marker for mature human MΦ, was significantly increased after PMA treatment, as shown in Figure 2A. Quantification analysis revealed levels exceeding 90% in PMA-induced MΦ.

### 3.2 The characterization of nab-PTX/MΦ

To determine the appropriate concentration of nab-PTX, assessing the viability of MΦ when exposed to the drug was crucial. The cytotoxicity of various concentrations of nab-PTX on MΦ was evaluated for 24 and 48 h. The results showed that lower concentrations of nab-PTX (5–30 μg/mL) did not have a significant effect on the viability of MΦ (Figures 2B, C). Conversely, higher concentrations (40–100 μg/mL) resulted in a notable decrease in viability. Consequently, it can be inferred that MΦ can effectively serve as carriers of nab-PTX at a concentration of 30 μg/mL. As shown in Figure 2D, the chemotaxis and migration assays were performed using a transwell system, where GBM cells were placed in the lower chamber and MΦ were placed in the upper chamber. The findings revealed no discernible difference between nab-PTX/MΦ and untreated MΦ (Figures 2E–G). The encapsulated nab-PTX was uniformly dispersed within MΦ as observed by LSCM. The membrane of MΦ stains with red fluorescence using DiI. As shown in Figure 2H, the green fluorescence signal of FITC-labeled nab-PTX could be clearly observed with MΦ, suggesting successful loading of nab-PTX into MΦ.

### 3.3 MΦ uptake and release of nab-PTX

**FIGURE 3:**
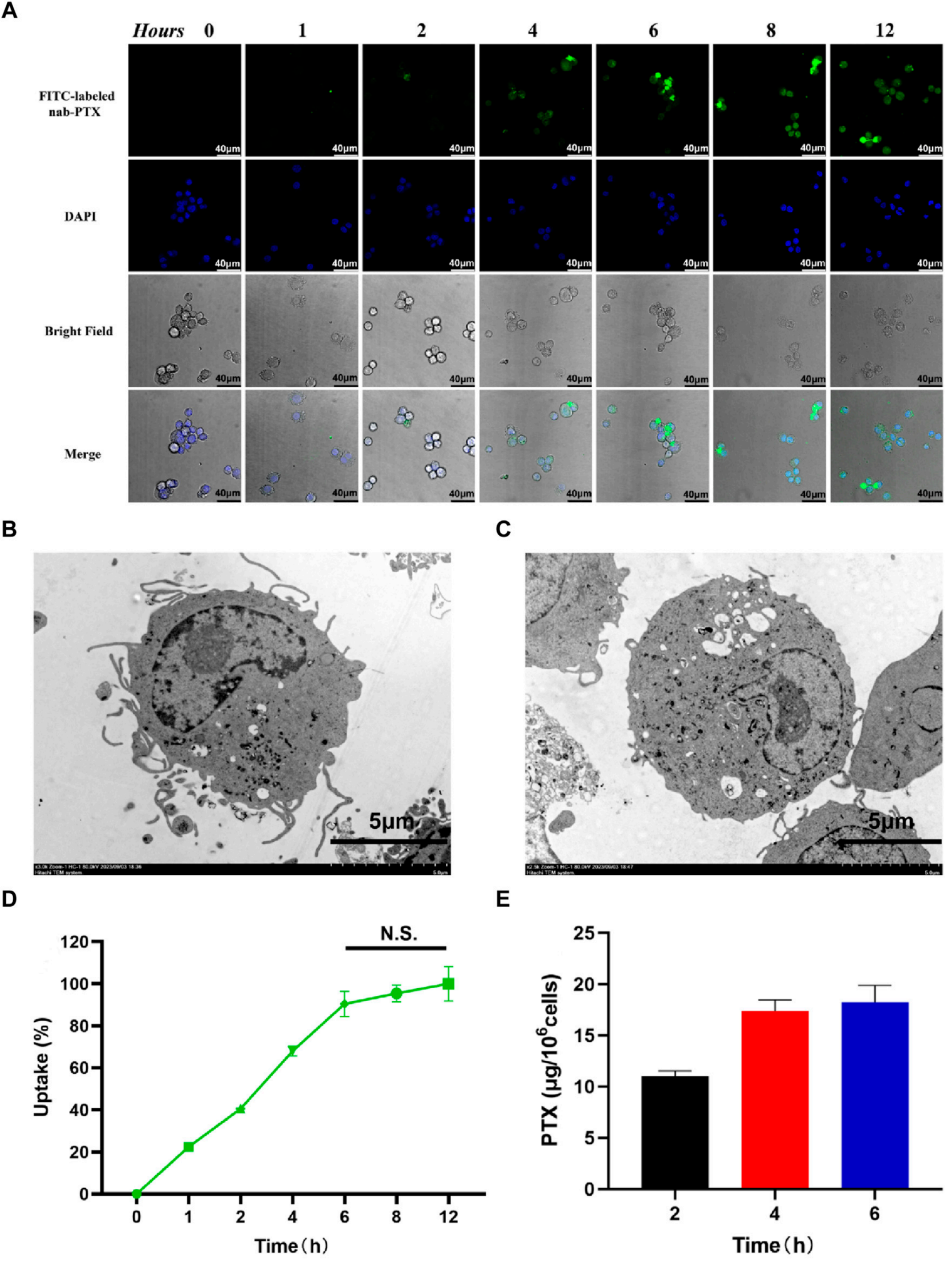
Uptake of nab-PTX by the MΦ. **(A)** The intracellular distribution of FITC-labeled nab-PTX (green) within MΦ treated with nab-PTX at different times. The nuclei of MΦ are stained by DAPI (blue). Scale bars represent 40 μm. **(B)** TEM images of MΦ without nab-PTX. **(C)** TEM images of nab-PTX/MΦ. Scale bars represent 5 μm. **(D)** The uptake of nab-PTX by MΦ, as indicated by an increase in fluorescence density over time. **(E)** The cell lysis buffers of nab-PTX/MΦ were collected and assayed using UPLC-MS.

As shown in Figure 3A, the uptake of nab-PTX by MΦ was observed to occur within 1 h of incubation. After MΦ uptake of nab-PTX, the cellular morphology of nab-PTX/MΦ remained unchanged compared to blank MΦ. Furthermore, the organelles (such as mitochondria and endoplasmic reticulum), cytomembrane, and cell nucleus of MΦ showed no signs of damage after loading with nab-PTX (Figures 3B, C). Semi-quantitative analysis of the immunofluorescent images: Uptake (%) = I1/Imax × 100% (Tao et al., 2013). As illustrated in Figure 3D, by 4 h, more than 50% of the MΦ contained varying levels of nab-PTX, with this percentage increased to 95% by 6 h, remaining constant until 12 h. These results indicated that the fluorescence density, serving as a measure of nab-PTX uptake, increases over time, demonstrating a time-dependent uptake of nab-PTX by MΦ. The fluorescence density at 6 h showed no statistical differences compared to 8 and 12 h, suggesting that the uptake of nab-PTX by MΦ had almost reached its saturation point at 6 h. Quantitative UPLC-MS analysis further confirmed a gradual increase in intracellular levels of PTX over time (Figure 3E).

**FIGURE 4:**
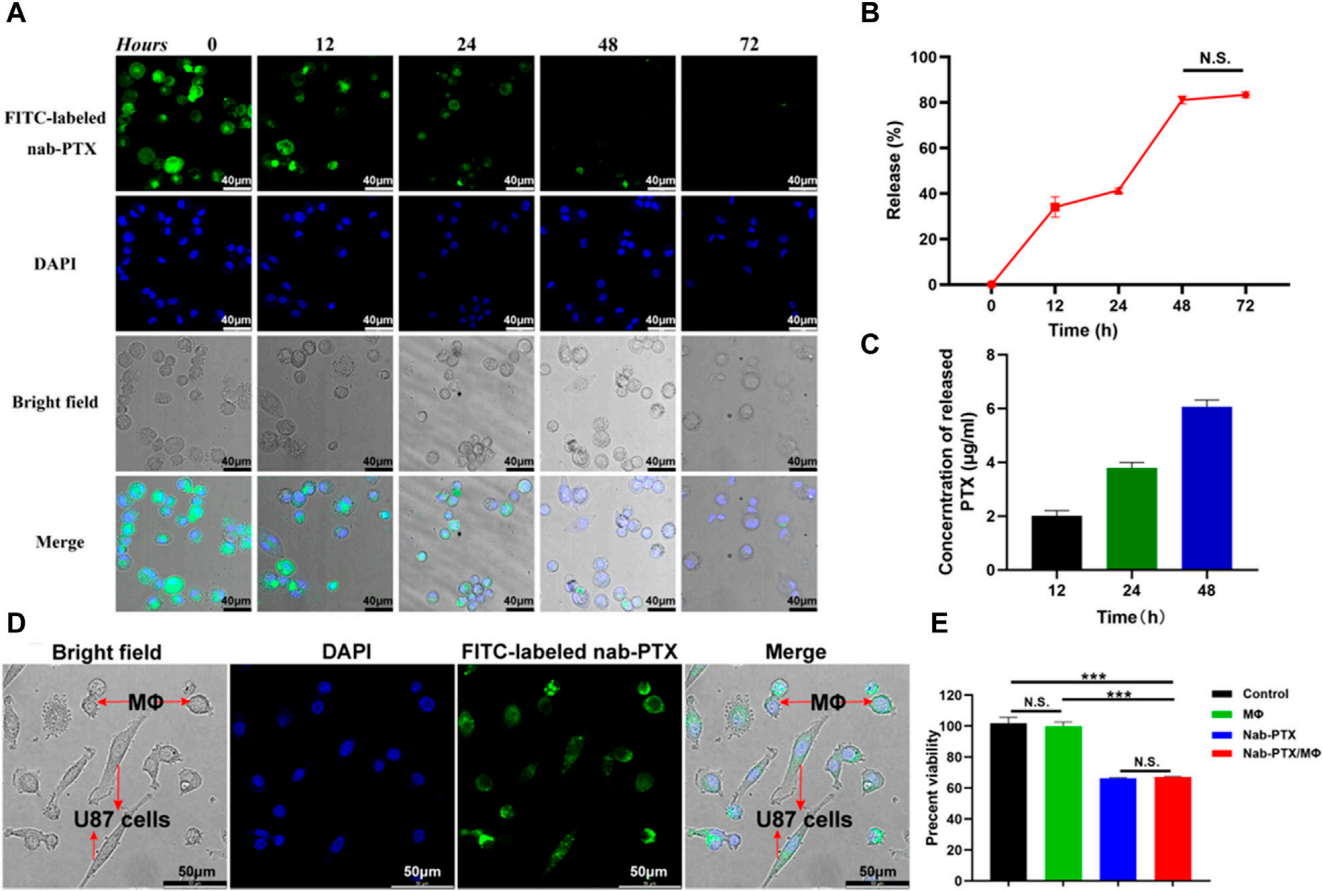
Release of nab-PTX from the nab-PTX/MΦ. **(A)** The intracellular distribution of FITC-labeled nab-PTX within MΦ at various time intervals after washout. The nuclei of MΦ are stained by DAPI (blue) Scale bars represent 40 μm. **(B)** The release of nab-PTX from MΦ, as evidenced by a decrease in fluorescence density over time. **(C)** The cultured nab-PTX/MΦ medium was collected and assayed by UPLC-MS. **(D)** Nab-PTX/MΦ were co-cultured with GBM cells for 24 h. Green fluorescence originated from FITC-labeled nab-PTX, while blue fluorescence represented nuclear counterstaining. Scale bars represent 50 μm. **(E)** The *in vitro* anti-tumor effects of nab-PTX released from nab-PTX/MΦ after incubation for 48 h.

Following phagocytosis, MΦ were able to release PTX. The evaluation of the released nab-PTX from MΦ were approximately conducted using the following formula: Release (%) = [1 – I1/Imax] × 100%, where Imax represents the average fluorescence intensity of images captured after 12 h of uptake, and I1 represents the average fluorescence intensity of images captured at various times (Tao et al., 2013). As shown in Figures 4A, B, the intracellular fluorescence density exhibited a rapid decrease subsequent to various time intervals. Approximately 40% of nab-PTX was released from MΦ into the extracellular environment after 24 h, and this percentage increased to 80% by 48 h. There were no significant differences between 48 h and 72 h, indicating that the release of nab-PTX by MΦ had nearly reached its limit at 48 h. Quantitative UPLC-MS analysis results showed that the release of nab-PTX from MΦ was consistent with a decrease in intracellular fluorescence density following various washout times (Figure 4C).

### 3.4 *In Vitro* cytotoxicity of nab-PTX/MΦ

We used FITC-labeled nab-PTX and MΦ to prepare nab-PTX/MΦ, subsequently co-cultured them with GBM cells for 24 h. As shown in Figure 4D, significant FITC-labeled nab-PTX fluorescence (green) was observed in both MΦ and GBM cells. This observation indicated the release of nab-PTX from nab-PTX/MΦ and its infiltration into GBM cells, To evaluate the anti-tumor effect of nab-PTX released from nab-PTX/MΦ, a CCK-8 assay was conducted on U87 cells after a 48-h incubation with the supernatant medium of nab-PTX/MΦ. Control groups consisted of untreated U87 cells and cells incubated with the supernatant medium of blank MΦ. The results demonstrated that nab-PTX released from nab-PTX/MΦ exhibited significant cytotoxicity against GBM cells, comparable to fresh nab-PTX (equivalent to 6 μg/mL of PTX) (Figure 4E). These findings indicated that the encapsulated nab-PTX could be released from nab-PTX/MΦ after a certain period of time and possessed superior toxicity against GBM cells.

### 3.5 Nab-PTX induces MΦ M1 polarization

**FIGURE 5:**
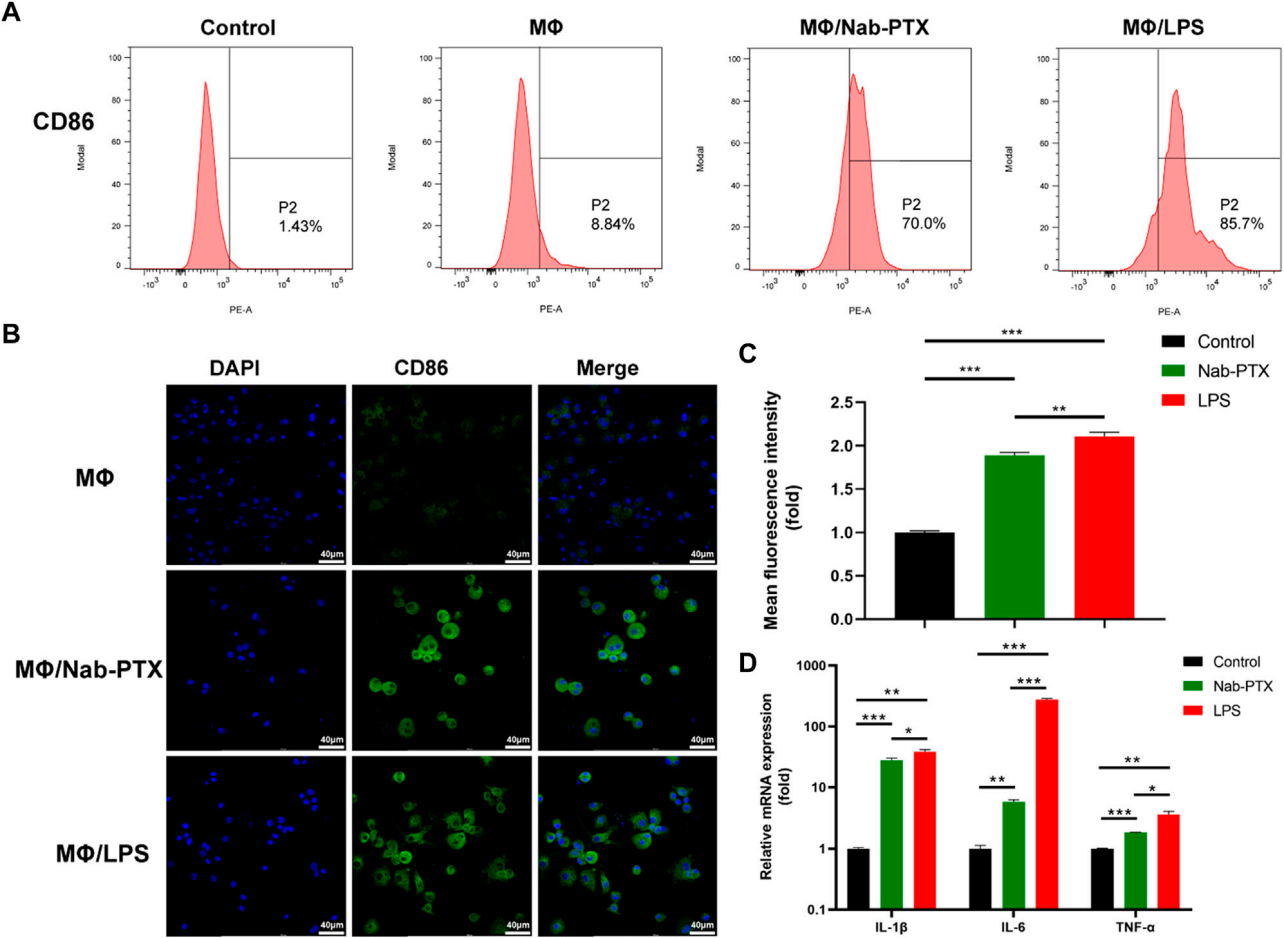
The expression of M1-phenotype markers in MΦ under different culture conditions. **(A)** The expression levels of CD86 in MΦ (treated with PBS, nab-PTX, and LPS, respectively) were determined by flow cytometry analysis. **(B)** Fluorescence images of MΦ (treated with PBS, nab-PTX, and LPS, respectively), stained with anti-CD86 (green). The nuclei of MΦ were stained with DAPI (blue). Scale bars represent 40 μm. **(C)** Fluorescence intensity of images was assessed using ImageJ software. **(D)** The mRNA expression levels of IL-1β, IL-6, and TNF-α were determined using real-time PCR.

PTX has been demonstrated to function as an LPS mimetic in activating the NF-κB pathway, which is involved in the regulation of MΦ M1 polarization (Cao et al., 2022). M1-phenotype MΦ are known to play a critical role in the immune response by producing various cytokines, which help to inhibit tumor cell proliferation and growth. Human M1-phenotype MΦ markers include CD86, IL-1β, IL-6, TNF-α (Yang et al., 2019; Yang et al., 2021; Zhao et al., 2021). Flow cytometry analysis revealed a significant increase in CD86 expression levels in the MΦ/nab-PTX and MΦ/LPS groups. As depicted in Figure 5A, the percentage of CD86-positive cells increased from 8.8% in the untreated group to 70% in the nab-PTX treated group, while the LPS-treated group increased to 85.7%. Immunofluorescence results also indicated that higher expression levels of CD86, a surface marker of the M1 phenotype, in the nab-PTX/MΦ and LPS/MΦ groups compared to the control MΦ group (Figures 5B, C). Real-time PCR analysis further confirmed the upregulation of M1-specific genes, such as IL-1β, IL-6, and TNF-α, after treatment with nab-PTX or LPS. As shown in Figure 5D, higher levels of IL-1β, IL-6, and TNF-α were detected in the nab-PTX/MΦ and LPS/MΦ compared to the untreated MΦ group. Furthermore, the levels of IL-1β, IL-6, and TNF-α in the LPS/MΦ group were found to be higher than those in the nab-PTX/MΦ group. The upregulation of the M1-specific marker CD86 and M1-specific genes confirms the polarization of MΦ to the M1-phenotype in response to nab-PTX or LPS treatment.

### 3.6 Construction of the microfluidic device and formation of GBM model

**FIGURE 6:**
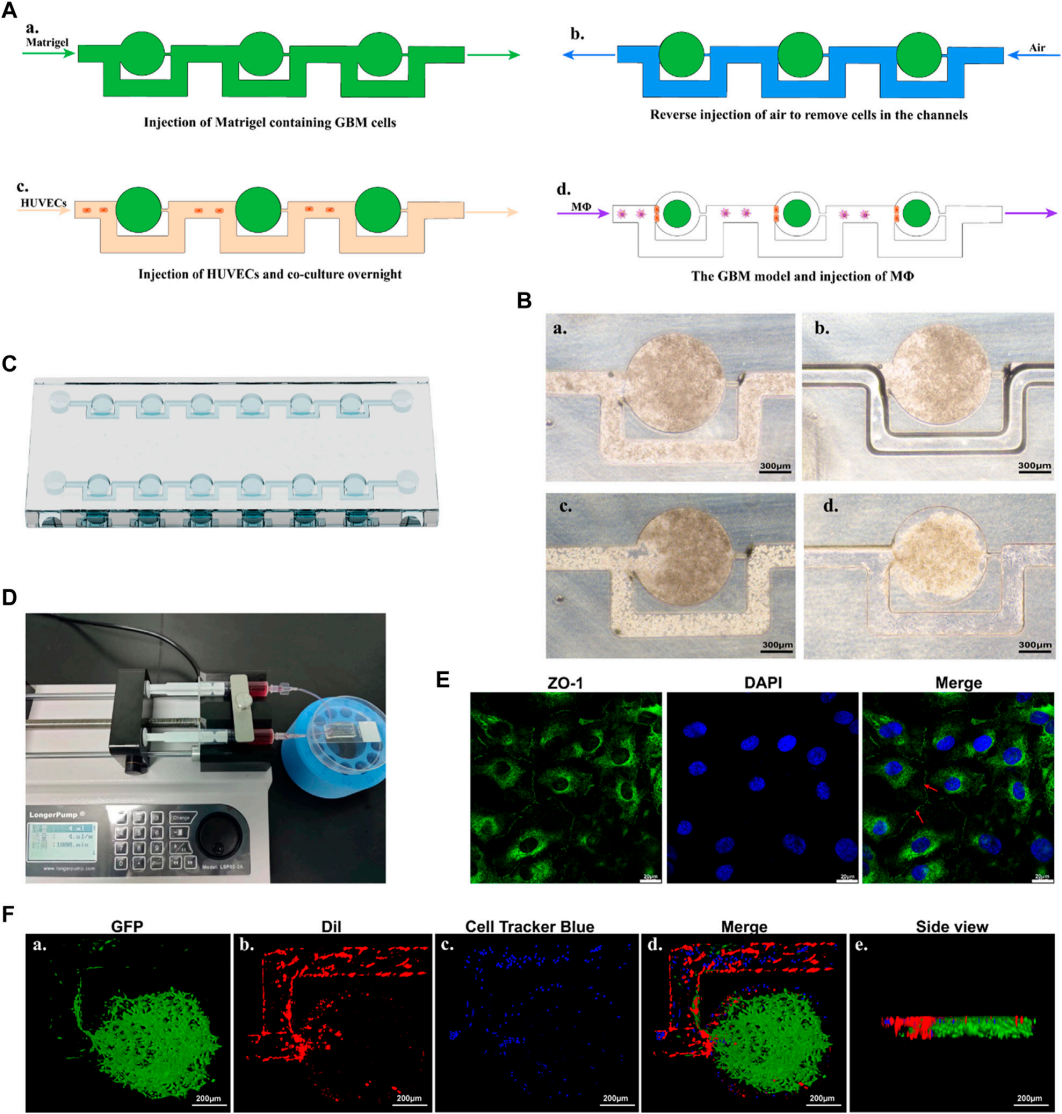
An overview of the construction process of the GBM model. **(A)** Schematic diagram of the microfluidic protocol. **(B)** Representative microscopy images of the construction process. a. Represents the injection of Matrigel containing GBM cells. b. Represents the reverse injection of air to remove cells in the channels. c. Represents the injection of HUVECs. d. Represents the GBM model cultured overnight. Scale bars represent 300 μm. **(C)** A schematic representation of the chip. **(D)** The microfluidic device with GBM models, connected to two syringes and a syringe pump. **(E)** Confocal images of ZO-1 and nucleus makers from HUVECs. Red arrow indicated the tight junction. Scale bars represent 20 μm. **(F)** 3D reconstruction of the GBM model using confocal images. Fluorescence green comes from U87-EGFP cells, fluorescence red originates from HUVECs, and fluorescence blue represents MΦ. Scale bars represent 200 μm.

In the initial step, Matrigel containing U87-MG cells was injected into the microfluidic chip (Figures 6Aa, Ba). Subsequently, air was injected in reverse to remove the cells present in the channels (Figures 6Ab, Bb). As the Matrigel containing cells approached an empty trapping site, it followed the path with the smallest fluidic resistance and entered a round chamber, where their morphology transformed into an oblate spheroid. Following the initial seeding of Matrigel, the cells bypassed previously trapped cells and occupied the next vacant chamber until all trapping sites were filled. Under these conditions, the cells remained in the round chambers, enabling spheroid incubation. Subsequently, HUVECs were injected into the chip, which was then positioned upright and left undisturbed overnight (Figures 6Ac, Bc). Successful formation of tumor spheroids with the BBB was achieved using the microfluidic chip (Figure 6Bd). The chip consisted of muti-central chambers and two side channels allowing controlled flow of media and cells (Figures 6C, D). Cells were introduced into the central chamber and allowed to aggregate into spheroids overnight. Subsequently, MΦ were introduced into the chip using a microfluidic-based perfusion system. Finally, the morphology of the spheroids was assessed using LSCM.

After constructing the model, immunostaining with ZO-1 antibody was performed to evaluate the functionalization of HUVECs. HUVECs in the model exhibited regular morphology, grew tightly, and were arranged in a cobblestone-like pattern. As shown in Figure 6E, under LSCM, positive expression of the ZO-1 protein (intercellular green fluorescence), an important protein marker for intercellular junctions, could be observed between HUVECs. This indicated a well-formed barrier with fairly tight junctions between endothelial cells.

The 3D visualization of the GBM model (green), the 3D HUVECs layer (red), and the perfused MΦ were provided in Figure 6F. U87-EGFP cells were used to produce the GBM spheroid according to the method described above. The findings showed that the GBM spheroid exhibited a flatter and cylindrical rather than the normal spherical shape, attributed to the constraints imposed by the microfluidic chip (Figure 6Fa). DiI was employed to label the cell membrane of HUVECs, and the cells were injected into the microfluidic chip according to the method described above. The results showed that a 3D layer of HUVECs formed, covering the microfluidic channels and positioned alongside the GBM spheroid, playing a significant role in mimicking the function of the endothelial barrier (Figure 6Fb). Cell Tracker Blue CMAC was used to label the MΦ, which were perfused into the microfluidic chip through a syringe connected to a syringe pump. The results showed that MΦ were able to across the HUVECs barrier and reach the GBM spheroid (Figure 6Fd).

The constructed device was finalized by connecting 0.8 mm (inner diameter) Teflon tubing to the inlet and outlet channels. The tube was sterilized with 75% ethanol and exposed to high-intensity UV light before use. The microfluidic chip was then connected to a 5 mL injection syringe filled with the appropriate media and linked to a syringe pump housed within a Perspex enclosure, which was maintained at a controlled temperature of 37°C.Medium was infused to incorporate interconnected microchannels and tissue chambers in the chip at a rate of 4 μL/min, enabling the continuous perfusion of GBM tissues with nutrient media.

### 3.7 Anti-tumor effects of nab-PTX/MΦ

**FIGURE 7:**
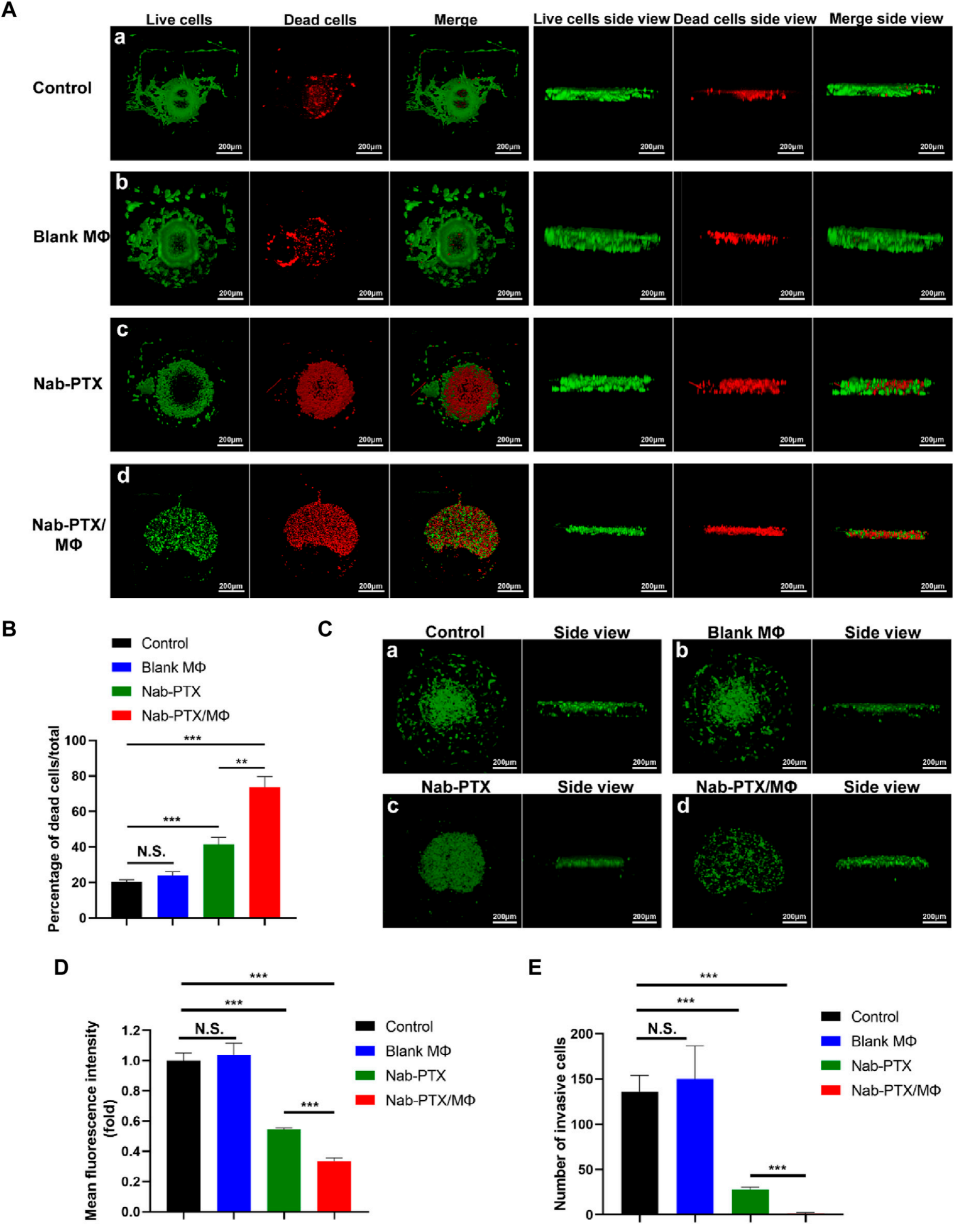
Evaluation of the anti-tumor effects of nab-PTX/MΦ in a microfluidic chip. **(A)** The GBM spheroid was stained with Calcein-AM after 48 h of various treatments, and then it was 3D reconstructed using LSCM. (a–d) Treatments with PBS, blank MΦ, nab-PTX, and nab-PTX/MΦ, respectively. Scale bars represent 200 μm. **(B)** 3D reconstruction of the U87-EGFP spheroid after 48 h of various treatments. (a–d) Treatments with PBS, blank MΦ, nab-PTX, and nab-PTX/MΦ, respectively. Scale bars represent 200 μm. **(C)** Percentage of dead cells relative to total cells. **(D)** Fluorescence intensity of U87-EGFP cell images was assessed using ImageJ software. **(E)** Quantitative analysis of the number of invasive GBM cells in the chip through confocal images.

In the microfluidic device, the effectiveness was determined by evaluating the viability of GBM cells through measuring the ratio of the number of dead cells (stained with PI) to the number of total cells (dead cells plus live cells, stained with calcein-AM) under different treatment groups (including the control group, blank MΦ group, nab-PTX group, and nab-PTX/MΦ group), as depicted in Figure 7A. There was no statistically significant difference in cell viability between the blank MΦ treatment group and the control group (Figure 7B). Under the treatment of nab-PTX alone and nab-PTX/MΦ, the average percentage of dead cells increased from 20% to 41% and 73%, respectively. In comparison to treating with nab-PTX alone, nab-PTX/MΦ exerted a more pronounced effect in inducing cell death. This could be attributed to the fact that nab-PTX is unable to fully penetrate the HUVECs barrier, and the induction of MΦ M1 polarization by nab-PTX may enhance its anti-tumor effectiveness. U87-EGFP cells express green fluorescent protein, which can be visualized as green-colored cells using LSCM (Figure 7C). This can also be used to monitor changes in cell morphology, proliferation, and apoptosis following various treatments. As shown in Figure 7D, the number of cells expressing green fluorescence also confirmed the effectiveness of nab-PTX/MΦ against GBM cells. There was no significant difference between the control group and the blank MΦ treatment group. After treatment with nab-PTX alone and nab-PTX/MΦ for 48 h, the number of cells expressing green fluorescence decreased significantly. The decrease in the nab-PTX/MΦ treatment group was more pronounced.

To evaluate the inhibitory effect of nab-PTX/MΦ on GBM invasion, the GBM model was treated with blank MΦ, nab-PTX alone, and nab-PTX/MΦ for 48 h in the microfluidic device, followed by an invasion assay. For the quantitative analysis of GBM cell invasion, we measured the number of invasive GBM cells. In this work, we utilized directional motility to evaluate the invasiveness of GBM cells. We defined the edge of a spheroid as the point where invasion begins and identified the migrating cells beyond this edge as invasive cells. The number of cells expressing green fluorescence was counted within their respective zones. As shown in Figure 7E, nab-PTX/MΦ exhibited a stronger inhibitory effect on the invasiveness of GBM cells compared to nab-PTX alone.

The formation of tumor spheroids is a critical step in the development and progression of cancer (Wang et al., 2020). In this work, we investigated the effects of various treatments on GBM spheroid formation. As shown in Figures 7A, B, the spheroids within the control group and the blank MΦ group in the microfluidic chip were agglomerating and spherical. Treating with nab-PTX/MΦ resulted in a significant decrease in spheroid formation. The GBM cell morphologies were appeared scattered, and the cells did not form 3D structures.

## 4 Discussion

MΦ are the predominant immune cell infiltrates within the tumor microenvironment (Zhang et al., 2019). Various potential strategies have been developed for utilizing MΦ to efficiently deliver small drug molecules or drug-loaded nanoparticles (Lee et al., 2019; Shields et al., 2020; Qiu et al., 2021; Zhu et al., 2023). MΦ loaded with therapeutic components can serve as excellent mediators for delivering these agents across the endothelial barrier and releasing drugs to specific targeted tissues. However, the acquisition of sufficient quantities of MΦ poses a challenge due to their status as terminally differentiated cells. Isolating primary MΦ directly from tissues is technically demanding and limited in yield. Immortalized monocytic cell lines, such as U937 monocytes, are frequently used as a source of MΦ, circumventing ethical limitations (Spalinger et al., 2020). Hence, in our investigation, U937-differentiated MΦ were chosen as vehicles for drug delivery and incubated with nab-PTX to form nab-PTX/MΦ. The specific marker for mature human MΦ, CD68, was highly expressed in U937-differentiated MΦ. Nab-PTX showed toxicity towards MΦ at the chosen concentration and exhibited a potential role in protecting MΦ biological functions. This effect could be attributed to the anti-tumor ability of PTX to inhibit cell proliferation (Song et al., 2023), which is absent in mature MΦ. Studies have demonstrated that tumor associated MΦ are able to uptake and accumulate nab-PTX, leading to the expression of M1 cytokines (Cullis et al., 2017; Li et al., 2021). However, the capability of human MΦ to load nab-PTX remains uncertain.

Our study revealed that MΦ have the capacity to internalize an approximate quantity 18 μg of PTX per 10^6^ cells, and PTX within the MΦ can be released into the surrounding media over time. Furthermore, our results showed that nab-PTX has no significant impact on the migratory and chemotactic abilities of MΦ. These findings were consistent with other MΦ-based drug delivery systems (Qiu et al., 2021; Zhu et al., 2023). Thus, due to the natural affinity of MΦ to tumor cells, nab-PTX/MΦ could migrate into the brain and achieve high PTX accumulation in tumor areas while reducing toxicity to surrounding healthy tissues. It is interesting that our engineered nab-PTX/MΦ exhibited M1 phenotype and nab-PTX loaded in MΦ may be the cause of phenotypic polarization. This could be attributed to the potential of nab-PTX to re-educate MΦ to M1 phenotype, which can directly inhibit tumors and reprogram the immunosuppressive tumor microenvironment to some extent. On the one hand, MΦ can serve as a means of delivering nab-PTX to target GBM tissues, thereby enhancing chemotherapy effectiveness. On the other hand, the combination of nab-PTX and MΦ can be used as a therapeutic tool with potential immunomodulatory effects.

To date, the efficacy of drug-loaded MΦ has mostly been assessed using two-dimensional cell cultures or animal models (Hou et al., 2020; Qiu et al., 2021; Mendanha et al., 2023). However, extensive use of animal models may be restricted due to their high cost, ethical concerns, and genetic background variations. *In vitro* generation of spheroids enables the simulation of proliferative conditions, metabolism, and concentration gradients of available substances within cancer cells, resembling *in vivo* environments (Fang et al., 2023). Based on the rate of cell proliferation, a solid tumor can be categorized into distinct zones, including the hypoxic, quiescent, and proliferative zones. Our study findings were consistent with this theory, as the majority of dead GBM cells were located in the core of the tumor spheroid.

Numerous studies have outlined methods for multicellular spheroid formation (Manfrin et al., 2019; Schuster et al., 2020). With the advancement of micro-nano fabrication, microfluidic technology has emerged as a valuable tool for addressing the challenges in traditional tumor spheroid cultures. Microfluidic devices offer the capability to exert precise control over small volumes by confining fluids to a scale that aligns with the size of tumor spheroids. This reduces sample consumption and minimizes microdomain effects, facilitating observation and analysis. Soft lithography, utilizing PDMS and a master mold, is commonly employed for producing microfluidic platforms, as demonstrated in this study. PDMS microchannels offer favorable biocompatibility, gas permeability, chemical inertness, optical transparency, and low autofluorescence, making them highly suitable for cell culture applications (Vasilescu et al., 2021).

However, there is still a lack of systematic study on the use of living macrophage-based carriers in such 3D microfluidic models. With this goal in mind, we developed a GBM model using a multichannel microfluidic device that mimics solid tumors and BBB. This model would realize a tumor-HUVEC coculture system. Through continuous perfusion, our model successfully maintained the viability and functionality of GBM cells. Meanwhile, the endothelial barrier structure in the model is intact. Additionally, we used a pipette to inject cells into the chip, exhibiting the convenience with which this microfluidic device can be utilized. The approach outlined in our study appears to offer a conveniently accessible method for generating tumor spheroids in laboratories, in contrast to droplet-based methods for tumor spheroid formation, which can be operationally complex and may require expensive additional equipment and specialized expertise that are not readily available in most laboratories (Lee et al., 2020).

To investigate the effectiveness of nab-PTX/MΦ on GBM cells, we compared all the GBM cells response under nab-PTX/MΦ, blank MΦ, and individual nab-PTX conditions, including cell viability, the number of the invasive GBM cells, and tumor spheroid formation ability. Qiu et al. (2021) showed that the natural cell membrane fusion capability of MΦ and the stimulation of the inflammatory microenvironment enable rapid delivery of drug-loaded nanoparticles into cancer cells. In another investigation into advanced gastric cancer, it was reported that the utilization of human neutrophils as carriers for nab-PTX exerted superior tumor suppression (Ju et al., 2019). We found that the nab-PTX/MΦ group showed a more pronounced impact on tumor inhibition compared to individual nab-PTX. This was mainly attributed to the tumoricidal properties of the M1 phenotype and its ability to penetrate the 3D HUVECs layer to target GBM cells. Hence, the MΦ-based delivery system for nab-PTX maybe emerge as a new potential strategy for the development of cell therapy and serve as an appealing alternative for traditional GBM chemotherapy.

The main novelty of this study lies in the evaluation of the MΦ-based drug delivery system using a microfluidic chip, where we verified its notable anti-tumor effects. This approach may serve as a valuable resource for evaluating the efficacy of the MΦ-based drug delivery system, potentially replacing the necessity of animal models. Additionally, the delivery of nab-PTX into GBM tissues based on MΦ is another significant concept of this study. It is worth noting that the concept of MΦ-based drug delivery in tumors or the central nervous system is not a novel idea (Evans et al., 2020; Li et al., 2021). However, in this work, we suggest that MΦ can function not only as carriers of drugs, but also as cells that can be modified to exhibit M1 features and act as effectors. This may contribute to the advancement of combined cell therapy and chemotherapy against GBM. At present, most MΦ-based nano-drug delivery systems are still in the preclinical research stage, and there are still many challenges in clinical translation and industrial development (Hou et al., 2020; Chen et al., 2023; Zhu et al., 2023; Ruan et al., 2024). Firstly, due to convenience of access, the same species allogeneic cells are commonly selected in current research. While macrophages are naturally occurring cells in the body, their immunogenicity, individual rejection reactions, and *in vivo* safety still need to be confirmed. For example, injection of exogenous macrophages may trigger immune responses in the body, leading to their clearance or activation of inflammatory reactions, thereby affecting their survival and function. Secondly, intravenous injection of MΦ will rapidly disappear from the circulation and accumulate in the lungs, then redistribute to organs such as the spleen and liver. In the future, research should continue to focus on prolonging circulation half-life, maintaining specific phenotypes *in vivo*, improving targeting capabilities, and improving strategies for nonspecific distribution. Thirdly, before clinical translation can be achieved, comprehensive evaluations of *in vivo* pharmacokinetic properties, administration methods, safety, and industrialization are necessary.

## 5 Conclusion

In sum, this study successfully developed an *in vitro* GBM model based on an excellent microfluidic platform, which was used to investigate the transportation of MΦ loaded with nab-PTX across the endothelial barrier for the treatment of GBM. The therapeutic effect of nab-PTX/MΦ on the GBM model shows the superior inhibition of GBM cell proliferation, spheroid formation, and migration, highlighting its potential as an effective therapeutic strategy for GBM. Furthermore, the utilization of the microfluidic chip to generate 3D cultures presents a significant opportunity for conducting *in vitro* preclinical studies on GBM and evaluating the efficacy of novel treatments for GBM.

## Data Availability

The original contributions presented in the study are included in the article/Supplementary Material, further inquiries can be directed to the corresponding authors.
