# Supplementary material for: Evaluation of nanoparticle albumin-bound paclitaxel loaded macrophages for glioblastoma treatment based on a microfluidic chip
Source: Front Bioeng Biotechnol. 2024 Mar 18;12:1361682. doi: 10.3389/fbioe.2024.1361682 (PMC10982336; doi:10.3389/fbioe.2024.1361682)
Supplement: Supplementary file 1 [file DataSheet1.PDF]

Supplementary Material

1 Supplementary Figures

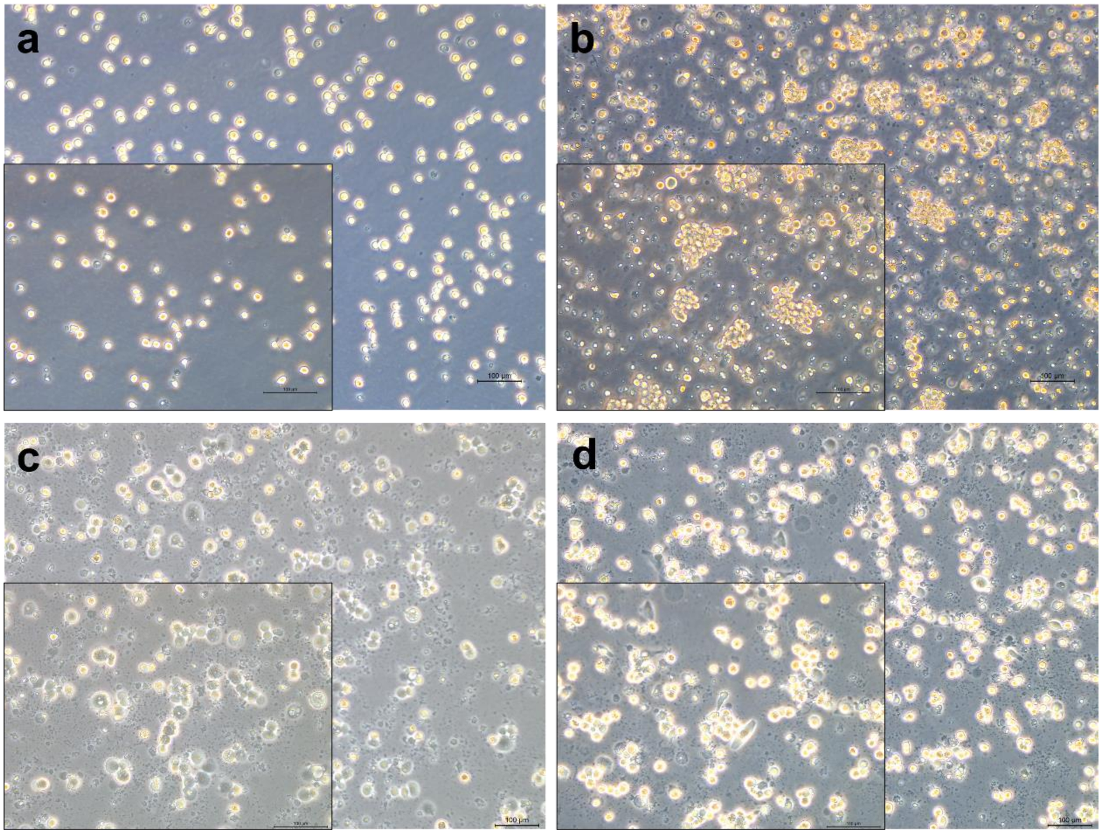

Supplementary Figure 1. (a) U937 cells. **(b)** U937 cells were treated with PMA for 24 h and differentiated into macrophages. **(c)** U937 cells differentiated macrophages were treated with LPS. **(d)** U937 cells differentiated macrophages were treated with nab-PTX.

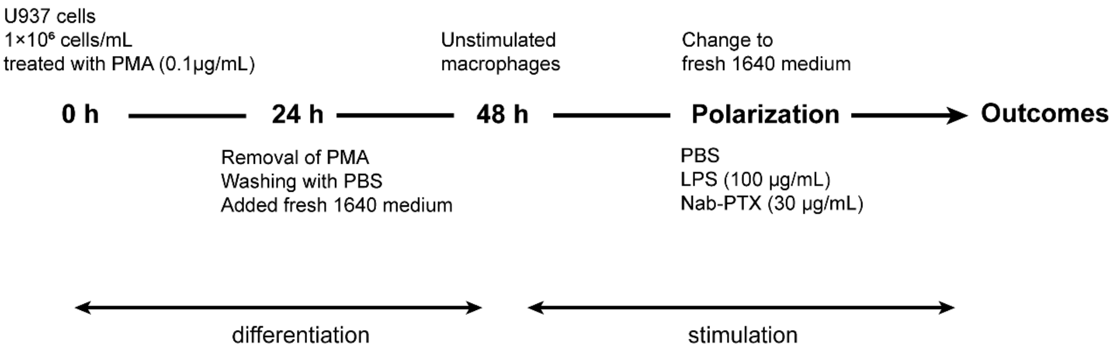

Supplementary Figure 2. Timeline of the experimental protocol used for PMA-induced macrophage differentiation and subsequent polarization in M1 conditions.
